# Expert Arguments for Trends of Psychiatric Bed Numbers: A Systematic Review of Qualitative Data

**DOI:** 10.3389/fpsyt.2021.745247

**Published:** 2021-12-24

**Authors:** Adrian P. Mundt, Sabine Delhey Langerfeldt, Enzo Rozas Serri, Mathias Siebenförcher, Stefan Priebe

**Affiliations:** ^1^Medical Faculty, Universidad Diego Portales, Santiago, Chile; ^2^Departamento de Neurología y Psiquiatría, Clínica Alemana de Santiago, Facultad de Medicina Clínica Alemana, Universidad del Desarrollo, Santiago, Chile; ^3^Department of Psychiatry and Mental Health, Hospital Clínico Universidad de Chile, Santiago, Chile; ^4^Department of Psychiatry and Psychotherapy Campus Mitte, Charité Universitätsmedizin Berlin, Berlin, Germany; ^5^Unit for Social and Community Psychiatry (WHO Collaborating Centre for Mental Health Service Development), Queen Mary University of London, London, United Kingdom

**Keywords:** psychiatric hospital beds, general hospital psychiatry, institutionalization, expert recommendation, consensus, inpatient, length of stay

## Abstract

**Introduction:** Mental health policies have encouraged removals of psychiatric beds in many countries. It is under debate whether to continue those trends. We conducted a systematic review of expert arguments for trends of psychiatric bed numbers.

**Methods:** We searched seven electronic databases and screened 15,479 papers to identify expert opinions, arguments and recommendations for trends of psychiatric bed numbers, published until December 2020. Data were synthesized using thematic analysis and classified into arguments to maintain or increase numbers and to reduce numbers.

**Results:** One hundred six publications from 25 countries were included. The most common themes arguing for reductions of psychiatric bed numbers were inadequate use of inpatient care, better integration of care and better use of community care. Arguments to maintain or increase bed numbers included high demand of psychiatric beds, high occupancy rates, increasing admission rates, criminalization of mentally ill, lack of community care and inadequately short length of stay. Cost effectiveness and quality of care were used as arguments for increase or decrease.

**Conclusions:** The expert arguments presented here may guide and focus future debate on the required psychiatric bed numbers. The recommendations may help policymakers to define targets for psychiatric bed numbers. Arguments need careful local evaluation, especially when supporting opposite directions of trends in different contexts.

## Introduction

### Rationale

Since the 1950's mental health services have undergone important transformations (1, 2). These included the development of community mental health care and the closure of psychiatric asylums and hospitals, which in most cases were built in the previous century. Reforms encouraged both reductions of psychiatric bed numbers and length of stay for psychiatric hospitalization, and promoted long-stay housing facilities in the community (3). At present, the reforms are still ongoing in many places. Substantial parts of inpatient psychiatric services have been removed and services have shifted toward community care (4–6). Reforms have not established a minimum or optimal number of psychiatric beds in order to assure balanced mental health systems, and it remains under debate whether to continue to remove psychiatric beds (7–10). Furthermore, research is still scarce on how many beds have actually been removed in the context of reforms and how these changes relate to other types of institutionalization (8).

The rates of psychiatric beds differ between countries and geographical regions (11), especially between different income groups (12). According to the WHO Mental Health Atlas (2017), the median number of psychiatric beds per 100,000 population is around 50 in high income countries (HIC), opposed to a rate of 7 in low and middle-income countries (LMIC). Residential care beds are almost inexistent in LMIC, whereas a median of 23 residential care beds per 100,000 population were reported for HIC (11).

The definition of psychiatric beds used by the Mental Health Atlas Project (11) incorporates short-stay and long-stay beds in psychiatric hospitals, beds in general hospital psychiatric units (GHPU), inpatient psychiatric services based in community settings and forensic inpatient units. This includes public and private facilities, psychiatric beds only for children and adolescents and other specific groups such as older adults. The definition excludes beds, which are exclusively used for the treatment of individuals with intellectual disability or substance use disorders, as well as facilities that exclusively provide rehabilitation and recovery services. The exact definition of beds in long-stay facilities causes difficulties (10, 13). The concept of psychiatric beds has undergone substantial changes over time (14, 15). This global variety of definitions limits international comparisons that aim to develop and optimize services (10, 16–18). A review assessing mental health plans of five English-speaking HIC showed that even in regions with the same official language, recommendations used variable nomenclature and mostly promoted a mix of inpatient and community services (16). It showed the need to define core mental health service components including specific resource targets in order to deliver more strategic clinical care.

Three different approaches have been proposed for estimating the required number of psychiatric beds (18). First, an empirical population health approach that estimates or calculates current and future psychiatric bed requirements for a specific catchment area based on epidemiological data considering current provision and quality of care (19). Secondly, expert consensus has been conducted in developed countries (20–22). Thirdly, a normative approach that assumes that different catchment areas with similar mental health and demographic profiles may require a similar number of psychiatric beds, so a well-functioning mental health system can be used as a model for other similar areas (18, 19).

We conducted a systematic review on expert opinions, arguments and recommendations for trends of psychiatric bed numbers.

## Methods

### Database Searches

The following seven databases were searched from their inception until December 27, 2020: PubMed, Embase Classic and Embase, PsycINFO and PsycIndex, Open Gray, Google Scholar, Global Health EBSCO and Proquest Dissertations. The search term used was “psychiatric AND hospital^*^ AND bed^*^” with no filters based on study types. We did not use any language restrictions. Since Google Scholar produces very high numbers of hits (>500,000) and sorts them by relevance, the search was limited to the first 561 hits. We restricted searches in PubMed, PsycINFO and PsycIndex to title and abstract. This helped us to assure that the psychiatric beds were a central topic of the articles rather than tangentially discussed. References and citations of articles retained in this study were reviewed for additional unidentified studies.

### Eligibility Criteria

We included studies that presented arguments, opinions, recommendations and suggestions for trends of psychiatric bed numbers. We excluded studies that only reported specific numbers of psychiatric beds. Studies providing purely numerical recommendations were excluded from this paper. Publications referring to beds or places in other mental health facilities such as residential facilities or day hospitals were also excluded.

### Data Collection Process

Literature screening was conducted by SD, Dr. ERS, and Dr. MS. Data were extracted independently by SD, Dr. ERS, and Dr. MS.

### Data Extraction

The following variables were extracted: year of publication, whether the expert recommendation referred to a local, national, regional or global area, the income group of the country for which the recommendation was made according to the World Bank classification (12), author's hypothesis or argument for the proposed change and recommendations. Multiple arguments were extracted, if present in the same publications.

### Data Analysis

The respective recommendations were a priori classified whether they dealt with acute care and short-stay on the one hand and long-stay on the other hand. When short- and medium-stay beds were reported in an aggregated format, they were classified as short-stay and when medium to long-stay beds were reported in an aggregated way, they were classified as long-stay. Furthermore, recommendations were grouped by suggestion to either reduce and maintain or increase psychiatric bed numbers. The arguments were analyzed using a thematic analysis approach with six steps (23), which is a qualitative analytic method used to identify, analyze and report codes, subthemes and themes for qualitative data. We used a theoretical rather than inductive thematic analyses approach (23), since the themes were sorted into the overarching a priori defined groups “reduce” or “increase- or maintain” psychiatric bed numbers. The themes were identified on a latent rather than semantic level (23). Findings from different publications were integrated in order to achieve an interpretation of the arguments that resulted in a higher level of scientific evidence, consistent with meta-synthesis (24). After familiarization with the texts, an open coding was developed and initial codes were determined. Then initial codes were grouped into different categories according to their similarities. In the third step, these categories were organized into themes. This implied the combination of codes into wider and overarching themes that adequately reflect the extracted information. Three authors subsequently examined this classification reorganizing the data and recoding the categories in a reiterative process between researchers and ensuring that categories were understood in the same way by all researchers. In the fifth step, a comprehensive analysis of the different themes was conducted ensuring that each one contributed to a better understanding of the data. For each category, a summary of the main ideas was derived from all included quotes. In the sixth and final step, the report was written allowing for a detailed description of the results. To ensure robustness, quotes were included in the description of the results to illustrate the descriptions.

The number of quoted recommendations per country and category were analyzed.

Critical appraisal tools typically aim to assess the quality of a study in particular. As quoted recommendations and expert arguments were not necessarily identical to the main objective of the respective studies, the methodology and overall quality of the publication were not suitable to assess the quality of the opinion. Thus, by consensus between the authors, we did not rank or differentiate the quality of the included recommendations or arguments. Nonetheless, in order to ensure validity of the results several quality criteria were included. First, triangulation was implemented meaning three complementary researchers from different backgrounds (psychology and psychiatry) participated in the data analysis. Secondly, theoretical validation was used to compare results with the scientific literature. Lastly, an iterative process was conducted. When a new code was added, the codification was read again in order to ensure that the initial classification was accurate and the extracted data were complete.

## Results

**Figure 1:**
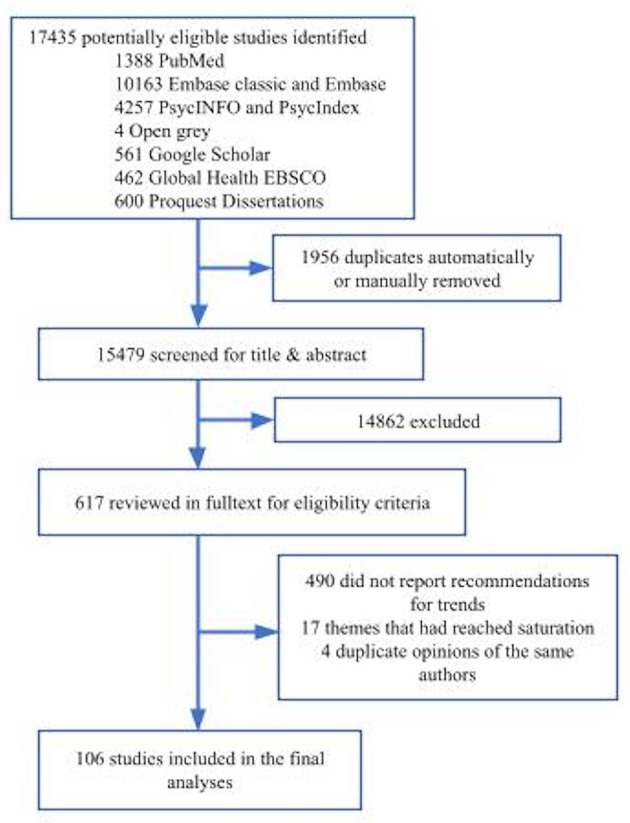
Flow diagram according to the preferred reporting items for systematic reviews and meta-analyses.

We followed the guidelines for Preferred Reporting Items of Systematic Reviews (PRISMA). The PRISMA flow chart is shown in Figure 1.

Expert arguments for trends of psychiatric bed numbers were reported in 106 publications (Table 1). Fifty studies made recommendations for short-stay beds, twelve for long-stay beds and 44 did not specify the length of stay. The studies were published between 1960 and 2020 from 25 different countries, including one study from Canada and Australia (10), one for global recommendations (WHO) (58) and one for the Eastern Mediterranean Region (EMR) (71). Ninety-two publications were from high-income countries (HIC), most from the United Kingdom and the United States. Fourteen studies referred to LMIC including Bhutan (68), Brazil (35, 53, 104), China (94), Ghana (74, 114), Moldova (88), Malawi (76), South Africa (55, 62), Uganda (103) and two publications referred to South America (8, 105). In 59 studies, recommendations were based on local or national epidemiological and demographical data. Recommendations based on expert consensus or opinion were found in 38 publications. Eleven recommendations were in line with a normative approach using institutional or governmental guidelines (14, 36, 53, 58, 79, 81, 88, 98, 103, 106). *Two* studies combined approaches (14, 88). Most expert arguments came from HIC (31) and only few (10) from LMIC (Table 2).

**Table 1:** Publications reporting recommendations and arguments for trends of psychiatric bed numbers sorted by length of stay.

**References**	**Year of publication**	**Country**	**Income group**	**International, national or local scope [catchment area or jurisdiction when local]**	**Type of publication**	**Length of stay**	**Approach to support recommendation**	**Suggested trend of psychiatric bed numbers**	**Argument for change per theme**
**Acute, short-stay and General Hospital psychiatric beds**									
Allison and Bastiampillai (25)	2015	Australia	HI	Local [South Australia]	Perspective	Acute	Expert opinion	Increase	Overcrowding and long waiting times in ED and early readmission
Baia Medeiros et al. (26)	2019	Canada	HI	Local [Toronto]	Original research	Acute (ED)	Original forecast	Increase	Hardships for patients and families, compromised safety and occurrence of serious incidents
Bastiampillai et al. (5)	2010	Australia	HI	Local [Adelaide]	Original research	Acute	Original estimate	Decrease	New care pathways and better integration of emergency departments, inpatient and outpatient services allow for further psychiatric bed removals
Bloom (27)	2015	US	HI	Local [Washington state]	Analysis & commentary	Acute (ED)	Expert opinion	Increase	Overcrowding and long waiting times in ED
Claudius (28)	2019	US	HI	National	Original research	Acute (pediatric, ED)	Original estimate	Do not increase	Bed reductions do not affect the quality of care in the system as a whole and has not shown negative effects
Davie (29)	2019	Australia	HI	National	Letter	Acute	Expert opinion	Increase	Increasing suicide rates, insufficient and ineffective community services
Dhillon (30)	2015	Australia	HI	Local [South Australia]	Correspondence	Acute	Expert opinion	Increase	Overcrowding and long waiting times in ED
Duthie (31)	2001	UK	HI	Local [Wales]	Correspondence	Acute (pediatric, ED)	Expert opinion	Increase	Lack of specialized psychiatric beds for children and adolescents
Early and Nicholas (32)	1971	UK	HI	Local [Bristol]	Original research	GHPU	Original estimate	Not do reduce	Increasing admission rates and waiting times, inappropriate admission due to lack of alternative care
Early and Nicholas (33)	1977	UK	HI	Local [Bristol]	Original research	GHPU	Original estimate	Decrease	Inappropriately long psychiatric inpatient care
Elpers and Crowell (34)	1982	US	HI	Global [developed countries]	Overview	Acute	Expert opinion	Not to eliminate	Insufficient and ineffective community service
Fagundes-Junior et al. (35)	2016	Brazil	UMI	Local [Rio de Janeiro]	Original research	GHPU	Original estimate	Stop bed reductions	Insufficient and ineffective community services
Flannigan et al. (36)	1994	UK	HI	Local [London]	Original research	Acute	Guidelines (normative)	Increase	Discharge to homelessness and shelters, increasing admission rates and waiting times
Ford, et al. (37)	2001	UK	HI	Local [North Birmingham]	Original research	Acute	Original estimate	Reduce	Lower cost of home treatment and outpatient care
Friebel et al. (38)	2019	UK	HI	National [England]	Original research	Acute	Original estimate	Increase	Short length of stay and premature discharge
Fulop et al. (39)	1996	UK	HI	Local [North and South Thames]	Original research	Acute	Original estimate	Not to increase	Inappropriately long psychiatric inpatient care, new care pathways and better integration of emergency departments, inpatient and outpatient services allow for further psychiatric bed removals
Harris (40)	1975	US	HI	Local [New York State]	Original research	GHPU	Original estimate	Reduce	Lower cost of home treatment and outpatient care, inappropriately long psychiatric inpatient care
Hatta et al. (41)	2010	Japan	HI	Local [Tokyo]	Original research	GHPU	Original estimate	Increase	High occupancy rates and overcrowding
Jones (42)	2013	UK	HI	Global	Original research	Acute	Original estimate	Increase	Implementation of community care complements, but does not replace inpatient care
Kalucy et al. (43)	2005	Australia	HI	Local [Adelaide]	Original research	Acute (ED)	Original estimate	Increase	Overcrowding and long waiting times in emergency departments
Kelly (44)	1998	Ireland	HI	Local [Northern Ireland]	Original research	Acute	Original estimate	Increase	High occupancy rates and overcrowding
Keown et al. (45)	2007	UK	HI	Local [Newcastle and North Tyneside]	Original research	Acute	Original estimate	Increase	Financial pressure on the mental health system has resulted in too many bed removals and underfunded inpatient care systems
La et al. (46)	2016	US	HI	Local [North Carolina]	Original research	Acute	Original estimate	Increase	Increasing admission rates and waiting times
Lamb and Weinberger (47)	2011	US	HI	National	Analysis & commentary	Acute	Expert opinion	Increase	Increasing detention rates due to lack of adequate and timely mental health treatments of persons with severe mental illnesses (and comorbid substance use disorders)
Laugharne et al. (48)	2016	UK	HI	Local [Cornwall]	Original research	Acute	Original estimate	Decrease	Inappropriately long psychiatric inpatient care, inpatient services are restrictive environments
Lee et al. (49)	2016	Hong Kong	HI	National	Letter	Acute	Expert opinion	Increase	Financial pressure on the mental health system has resulted in too many bed removals and underfunded inpatient care systems, high occupancy rates and overcrowding
Lelliott (50)	1996	UK	HI	National [England]	Original research	Short-stay (admission)	Expert opinion	Increase	Increasing admission rates and waiting times, limited post-discharge support in the community
Lelliott (51)	2006	UK	HI	National [England]	Short report	Acute	Expert opinion	Increase	Lack of specialized psychiatric beds for children and adolescents, hardships for patients and families, compromised safety and occurrence of serious incidents
Lippert et al. (52)	2016	US	HI	National	Original research	Acute (ED)	Original estimate	Increase	Overcrowding and long waiting times in emergency departments, insufficient and ineffective community services
Loch et al. (53)	2016	Brazil	UMI	Global [South America]	Review	GHPU	Guidelines (normative)	Increase	Insufficient and ineffective community services
Long (54)	2015	Australia	HI	Local	Correspondence	Acute	Expert opinion	Increase	Increasing admission rates and waiting times
Lund and Flisher (55)	2006	South Africa	UMI	National	Original research	Acute	Original estimate	Increase	Need for the development of integrated health care systems with decentralized inpatient care capacities
MacDonald et al. (56)	1999	New Zealand	HI	Local (Wellington)	Original research	Acute	Original estimate	Increase	High occupancy rates and overcrowding
Malcolm (57)	1989	New Zealand	HI	National	Original research	Short-stay (admission)	Original estimate	Decrease	Lower cost of home treatment and outpatient care, inpatient psychiatric bed capacity and availability generates utilization and coercive treatments
Morris et al. (58)	2012	Global (WHO)	HI & LMI	Global (184 countries)	Original research	GHPU	Guidelines (normative)	Balance bed reduction with increase of community care	Sub-groups of people with severe mental illnesses Are still in need of psychiatric inpatient beds
Munk-Jorgensen (59)	1999	Denmark	HI	National	Original research	Acute	Expert opinion	Do not reduce	High occupancy rates and overcrowding, increasing admission rates and waiting times, increasing suicide rates criminalization of mentally ill
Nicks and Manthey (60)	2012	US	HI	Local (61)	Original research	Acute (ED)	Original estimate	Increase	Overcrowding and long waiting times in emergency departments
Niehaus et al. (62)	2008	South Africa	UMI	Local [Western Cape Province]	Original research	Acute	Original estimate	Increase	Early readmission rates
Nordstrom et al. (63)	2019	US	HI	National	Original research	Acute (ED)	Expert opinion	Increase	Financial disincentives and unfair reimbursement practice have led to lower numbers of psychiatric beds than actually needed
O'Doherty (64)	1998	Ireland	HI	Local [NR]	Original research	Acute	Original estimate	Reduce	Implementation of day hospital services and home treatment teams allow for greater concentration of inpatient resources on most severely ill patients, leading to cost savings
O'Reilly and Chamberlaine (65)	2000	Canada	HI	National	Letter	Acute	Expert opinion	Do not reduce	Increasing admission rates and waiting times, hardships for patients and families, compromised safety and occurrence of serious incidents
O'Neil et al. (66)	2016	US	HI	Local [Rochester, Minnesota]	Original research	Acute (ED)	Original estimate	Increase	Overcrowding and long waiting times in emergency departments, risk of transfer outside patients' local community for care, hardships for patients and families, compromised safety and occurrence of serious incidents
Parker et al. (67)	2015	Australia	HI	National	Correspondence	Acute	Expert opinion	Increase	Implementation of community care complements, but does not replace inpatient care
Pelzang (68)	2012	Bhutan	LMI	Local [Thimphu]	Original research	Short-stay (admission)	Original estimate	Increase	Increasing admission rates and waiting times
Powell et al. (69)	1995	UK	HI	Local [Greater London]	Original research	Short-stay (admission)	Original estimate	Increase	High occupancy rates and overcrowding
Prins (70)	2011	US	HI	National	Short report	Acute	Expert opinion	Increase	Sub-groups of people with severe mental illnesses are still in need of psychiatric inpatient beds, criminalization of mentally ill
Saraceno et al. (71)	2015	EMR		Regional [EMR]	Original research	GHPU	Guidelines (normative)	Reduce	Reduced number of long-stay patients allows for further bed removals
Shumway et al. (6)	2012	US	HI	Local [San Francisco]	Original research	Acute	Original estimate	Reduce	Bed reductions do not affect the quality of care in the system as a whole and has not shown negative effects
Thomas (72)	2003	US	HI	National	Editorial	Acute (pediatric, ED)	Expert opinion	Increase	Short length of stay and premature discharge, lack of specialized psychiatric beds for children and adolescents
Tyrer et al. (73)	2017	UK	HI	National	Correspondence	Acute	Expert opinion	Increase	Short length of stay and premature discharge, risk of transfer outside patients' local community for care, hardships for patients and families, compromised safety and occurrence of serious incidents, increase in involuntary admissions due to lack of timely voluntary admission at an earlier stage of illness, implementation of community care complements, but does not replace inpatient care
**Medium—and long-stay**									
Akpalu et al. (74)	2010	Ghana	LI	National	Original research	Long-stay	Expert consensus	Increase	High occupancy
Allison et al. (75)	2018	Australia	HI	Local [Victoria]	Commentary	Long-stay	Expert opinion	Increase	Severe emotional and physical harm to patients, families and communities
Barnett et al. (76)	2019	Malawi	LI	Local [Lilongwe]	Original research	Long-stay	Original estimate	Increase	Need for the development of integrated health care systems with decentralized inpatient care capacities
Giel (77)	1986	Netherlands	HI	National	Original research	Long-stay	Original estimate	Not to reduce	Sub-groups of people with severe mental illnesses are still in need of psychiatric inpatient beds
Hailey (78)	1971	UK	HI	Local [Camberwell, England]	Original research	Long-stay	Original forecast	Reduce	Reduced number of long-stay patients allows for further bed removals
Holloway et al. (79)	1999	UK	HI	Local [East Lambeth and South Southwark, London]	Original research	Long-stay	Guidelines (normative)	Decrease	Reduced number of long-stay patients allows for further bed removals, bed reductions lead to better use of existing community care
Kim (80)	2017	Korea	HI	National	Commentary	Long-stay	Expert opinion	Decrease	Follow global trends of psychiatric bed reductions in most of the developed countries
Lelliott and Wing (14)	1994	UK	HI	Global [England & Wales, Scotland and Northern Ireland]	Original research	Medium- and long-stay	Guidelines (normative) and Expert consensus	Not to reduce	Limited post-discharge support in the community
Lesage and Tansella (81)	1993	Canada	HI	Global [Canada & Italy]	Original research	Long-stay	Guidelines (normative)	Reduce	Reduced number of long-stay patients allows for further bed removals
Madianos (82)	2002	Greece	HI	National	Original research	Long-stay	Original estimate	Reduce	Bed reductions, while maintaining personnel, improves inpatient care conditions
Okayama et al. (83)	2020	Japan	HI	National	Original research	Long-stay	Original forecast	Reduce	Reduced number of long-stay patients allows for further bed removals, follow global trends of psychiatric bed reductions in most of the developed countries
Sisti et al. (84)	2015	US	HI	National	Viewpoint	Long-stay	Expert opinion	Increase	Need for the development of safe, modern and humane asylums that provide long-term residential care for people with severe mental illnesses
**Non-specified length of stay**									
Allison et al. (85)	2017	Australia	HI	Global [Australia, UK and Canada]	Correspondence	Inpatient	Expert opinion	Increase	Increased detentions and bed pressure
Bowersox et al. (86)	2013	US	HI	National [VHA]	Original research	Inpatient	Original estimate	Do not change	Short LOS and premature discharge, need for the development of safe, modern and humane asylums that provide long-term residential care for people with severe mental illnesses
Dazzan and Barbui (87)	2015	UK	HI	National	Editorial	Inpatient	Expert's opinion	Not to increase	High occupancy, increase in involuntary admissions due to lack of timely voluntary admission at an earlier stage of illness, limited post-discharge in the community
De Vetten et al. (88)	2019	Moldova	LMI	National	Original research	Inpatient	Guidelines (normative) and Expert consensus	Reduce	New care pathways and better integration of emergency departments, inpatient and outpatient services allow for further psychiatric bed removals, follow global trends of psychiatric bed reductions in most of the developed countries
Fioritti et al. (89)	1997	Italy	HI	Local [Emilia-Romagna]	Original research	Inpatient	Original estimate	Reduce	New care pathways and better integration of emergency departments, inpatient and outpatient services allow for further psychiatric bed removals
Fisher et al. (90)	1996	US	HI	Local [Massachusetts]	Original research	Inpatient	Original estimate	Reduce	Hospital bed numbers should be reduced to serve the most severely ill patients
Forchuk et al. (91)	2006	Canada	HI	Local [London, Ontario]	Original research	Inpatient	Original estimate	Not to reduce	Short length of stay and premature discharge, discharge to homelessness and shelters
Forrester et al. (92)	2013	UK	HI	Local [London]	Original research	Inpatient (forensic)	Original estimate	Increase	Delays in transferring individuals with mental disorders in the criminal justice system to hospitals due to inpatient bed shortage
Geller and Biebel (93)	2006	US	HI	National	Original research	Inpatient (pediatric)	Original estimate	Not to reduce	Lack of specialized psychiatric beds for children and adolescents, increasing suicide rates, criminalization of mentally ill
Geng et al. (94)	2020	China	UMI	National	Original research	Inpatient (pediatric)	Original estimate	Increase	Lack of specialized psychiatric beds for children and adolescents
Goldman and Keller (95)	1978	US	HI	National	Original research	Inpatient	Original estimate	Reduce	Low inpatient occupancy rates
Guaiana et al. (10)	2019	Australia & Canada	HI	Global	Correspondence	Inpatient	Expert opinion	Do not reduce	High occupancy rates and overcrowding, overcrowding and long waiting times in emergency departments, early readmission rates, discharge to homelessness and shelters, criminalization of mentally ill
Hartvig and Kjelsberg (96)	2009	Norway	HI	National	Original research	Inpatient	Original estimate	Increase	Criminalization of mentally ill
Hollander et al. (97)	1996	UK	HI	Local [Greater London, England]	Letter	Inpatient	Expert opinion	Increase	High occupancy rates and overcrowding, short length of stay and premature discharge, hardships for patients and families, compromised safety and occurrence of serious incidents
Hume and Rudin (98)	1960	US	HI	Local [California]	Original research	Inpatient	Guidelines (normative)	Increase	Lack of specialized psychiatric beds for children and adolescents
Jeppesen et al. (99)	2016	Denmark	HI	National	Original research	Inpatient (schizophrenia)	Original estimate	Increase	Increasing admission rates and waiting times, lack of available inpatient beds and treatment for schizophrenia patients
Johnson (100)	2011	UK	HI	National [England & Wales]	Short report (opinion)	Inpatient	Expert opinion	Do not increase	Bed reductions reduce reliance on inpatient services
Kaltiala-Heino et al. (101)	2001	Finland	HI	Local (northern Finland)	Original research	Inpatient	Original estimate	Increase	Short length of stay
Keown et al. (102)	2019	UK	HI	National [England]	Original research	Inpatient (forensic)	Original estimate	Increase	Criminalization of mentally ill
Kigozi et al. (103)	2010	Uganda	LI	National	Original research	Inpatient	Guidelines (normative)	Increase	High occupancy rates and overcrowding
Kilsztajn et al. (104)	2008	Brazil	UMI	National	Original research	Inpatient	Original estimate	Decrease	Reduce resources for inpatient care to develop outpatient care
Lamb (105)	2015	Latin America	HI & LMI	South America	Editorial	Inpatient	Expert opinion	Increase	Criminalization of mentally ill
Lawrence et al. (106)	1991	England	HI	Local [Kidderminster District]	Original research	Inpatient	Guidelines (normative)	Maintain	Sub-groups of people with severe mental illnesses are still in need of psychiatric inpatient beds, implementation of community care complements, but does not replace inpatient care
Lelliott and Audini (107)	2003	UK	HI	Local [seven local authority areas, England]	Original research	Inpatient (forensic, young men)	Original estimate	Increase	Early readmission rates, increasing detention rates due to lack of adequate and timely mental health treatments of persons with severe mental illnesses (and comorbid substance use disorders)
MacDonald (108)	1989	Italy	HI	Local (Rome)	Original research	Inpatient	Expert consensus	Do not reduce	Insufficient and ineffective community services
Mundt et al. (8)	2015	South America	LMI	Global	Original research	Inpatient	Original estimate	Increase	Criminalization of mentally ill
Munk-Jorgensen and Mortensen (109)	1993	Denmark	HI	National	Short report	Inpatient (schizophrenia)	Expert opinion	Decrease is possible without negative effects	Decrease in first-ever admission rates of schizophrenia
Nilsson and Lögdberg (110)	2008	Sweden	HI	Local [Malmö]	Original research	Inpatient (schizophrenia)	Original estimate	Increase	Lack of available inpatient beds and treatment for schizophrenia patients
Nome and Holsten (111)	2011	Norway	HI	Local [Hordaland County]	Original research	Inpatient	Original estimate	Do not reduce	Implementation of community care complements, but does not replace inpatient care
Nordentoft et al. (112)	1996	Denmark	HI	Local [Copenhagen]	Original research	Inpatient	Original estimate	Increase	Implementation of community care complements, but does not replace inpatient care
O'Neil et al. (113)	2002	Ireland	HI	National	Original research	Inpatient (forensic)	Original estimate	Increase	Criminalization of mentally ill
Ose et al. (9)	2018	Norway	HI	National	Original research	Inpatient	Original estimate	Do not reduce	Insufficient and ineffective community services
Roberts et al. (114)	2014	Ghana	LMI	National	Original research	Inpatient	Original estimate	Increase	High occupancy rates and overcrowding
Rothbard et al. (115)	1998	US	HI	Local [Philadelphia]	Original research	Inpatient	Original estimate	Increase	Higher total health care system costs due to lack of beds (queuing in General Hospitals)
Sasaki (116)	2012	Japan	HI	Local	Original research	Inpatient	Expert opinion	Reduce	Economic incentives for inadequately long inpatient bed use
Someya et al. (117)	2004	Japan	HI	Local [Niigata Prefecture]	Original research	Inpatient (schizophrenia)	Original forecast	Reduce	Trend analyses show less psychiatric bed needs of schizophrenia patients
Svab et al. (118)	2006	Slovenia	HI	National	Short report	Inpatient	Expert opinion	Increase	Long waiting lists for outpatient services
Tim (75)	2013	UK	HI	National	Editorial	Inpatient (forensic)	Expert opinion	Increase	Increasing detention rates due to lack of adequate and timely mental health treatments of persons with severe mental illnesses (and comorbid substance use disorders)
Torrey et al. (119)	2012	US	HI	National	Report (Treatment Advocacy Center)	Inpatient	Expert opinion	Do not reduce	Overcrowding and long waiting times in emergency departments, sub-groups of people with severe mental illnesses are still in need of psychiatric inpatient beds, criminalization of mentally ill
Trieman and Leff (120)	1996	UK	HI	Local [North London]	Original research	Inpatient	Original estimate	Reduce	Inpatient services are restrictive environments
Weller and Weller (121)	1988	England	HI	Local [London]	Original research	Inpatient (forensic)	Expert opinion	Increase	Criminalization of mentally ill
Worrall and O'Herlihy (122)	2001	UK	HI	National	Original research	Inpatient (pediatric)	Expert consensus	Increase	Overcrowding and long waiting times in emergency departments, lack of specialized psychiatric beds for children and adolescents
Yoon and Bruckner (123)	2009	US	HI	National	Original research	Inpatient	Original estimate	Do not reduce	Increasing suicide rates, insufficient and ineffective community services
Yoon et al. (124)	2013	US	HI	Local [King County, Washington]	Original research	Inpatient (forensic, SMI)	Original estimate	Do not reduce	Increasing detention rates due to lack of adequate and timely mental health treatments of persons with severe mental illnesses (and comorbid substance use disorders)

*ED, emergency department; GHPU, general hospital psychiatric units; LOS, length of stay; HI, High-Income; SMI, severely mentally ill patients; UK, United Kingdom; USA, United States of America; UMI, Upper Middle-Income; LMI, Low- and Medium-Income; VHA, veterans health administration*.

**Table 2:** Number of expert arguments per theme and country.

**Themes**	**World Bank Income classification**																													
	**High-Income Countries (HIC)**																			**Low- and Middle-Income Countries (LMIC)**										
	**Australia**	**Canada**	**Denmark**	**Finland**	**Greece**	**Hong Kong**	**Ireland**	**Italy**	**Japan**	**Korea**	**Netherlands**	**NZ**	**Norway**	**UK**	**US**	**Slovenia**	**Sweden**	**Global**	**Total HIC**	**Brazil**	**Buthan**	**China**	**Ghana**	**Malawi**	**Moldova**	**Uganda**	**South Africa**	**South America**	**Total LMIC**	**Total HIC and LMIC**
**Expert arguments to reduce psychiatric bed numbers**																														
1.1. Cost effectiveness																														
1.1.1. Lower overall cost of home-based treatment compared with inpatient services												1		1	1				**3**										**0**	**3**
1.1.2. Implementation of a day hospital service and home treatment teams allows for greater concentration of inpatient resources on most severely ill patients, leading to cost savings							1												**1**										**0**	**1**
1.1.3. Reduce resources for inpatient care to develop outpatient care																			**0**	1									**1**	**1**
1.2. Inappropriate use of inpatient care																														
1.2.1. Inappropriately long psychiatric inpatient care							1		1					3	1				**6**										**0**	**6**
1.2.2. Reduced number of long-stay patients allows for further psychiatric bed removals		1							1					2				1	**5**										**0**	**5**
1.2.3. Inpatient psychiatric bed capacity and availability generates utilization and coercive treatments												1							**1**										**0**	**1**
1.2.4. Economic incentives for inadequately long inpatient bed use									1										**1**										**0**	**1**
1.3. Bed reductions lead to better use and development of existing community care														1					**1**										**0**	**1**
1.4. Quality of care is maintained or improved with less beds																														
1.4.1. Bed reductions, while maintaining personnel, improves inpatient care conditions					1														**1**										**0**	**1**
1.4.2. Bed reductions do not affect the quality of care in the system as a whole and has not shown negative effects															2				**2**										**0**	**2**
1.5. Less psychiatric bed needs																														
1.5.1. Trend analyses show less psychiatric bed needs of schizophrenia patients									1										**1**										**0**	**1**
1.5.2. Decrease in first-ever admission rates of schizophrenia			1																**1**										**0**	**1**
1.5.3. Low inpatient occupancy rates															1				**1**										**0**	**1**
1.6. Inpatient services are restrictive environments														2					**2**										**0**	**2**
1.7. New care pathways and better integration of emergency departments, inpatient and outpatient services allow for further psychiatric bed removals	1							1						1					**3**						1				**1**	**4**
1.8. Follow global trends of psychiatric bed reductions in most of the developed countries									1	1									**2**						1				**1**	**3**
1.9. Bed reductions reduce reliance on inpatient services														1					**1**										**0**	**1**
1.10. Hospital bed numbers should be reduced to serve the most severely ill patients															1				**1**										**0**	**1**
Total	**1**	**1**	**1**	**0**	**1**	**0**	**2**	**1**	**5**	**1**	**0**	**2**	**0**	**11**	**6**	**0**	**0**	**1**	**33**	**1**	**0**	**0**	**0**	**0**	**2**	**0**	**0**	0	**3**	**36**
**Expert arguments to increase or maintain psychiatric bed numbers**																														
2.1. Lack of beds for financial pressure																														
2.1.1. Financial pressure on the mental health system has resulted in too many bed removals and underfunded inpatient care systems						1								1					**2**										**0**	**2**
2.1.2. Financial disincentives and unfair reimbursement practice have led to lower numbers of psychiatric beds than actually needed															1				**1**										**0**	**1**
2.2. Higher total health care system costs due to bed closures (queuing in General Hospitals)															1				**1**										**0**	**1**
2.3. High demand of psychiatric beds																														
2.3.1. High occupancy rates and overcrowding			1			1	1		1			1		2				1	**8**				2			1			**3**	**11**
2.3.2. Increasing admission rates and waiting times	1	1	2											3	1				**8**		1								**1**	**9**
2.3.3. Overcrowding and long waiting times in emergency departments	3													1	5			1	**10**										**0**	**10**
2.4. Inadequately short length of stay																														
2.4.1. Short length of stay and premature discharge		1		1										3	2				**7**										**0**	**7**
2.4.2. Revolving door effect: Early readmission rates	1		1											1				1	**4**								1		**1**	**5**
2.5. Lack of specialized psychiatric beds for children and adolescents														3	3				**6**			1							**1**	**7**
2.6. Lack of locally available beds																														
2.6.1. Need for the development of integrated health care systems with decentralized inpatient care capacities																			**0**					1			1		**2**	**2**
2.6.2. Risk of transfer outside patients' local community for care														1	1				**2**										**0**	**2**
2.7. Lack of beds compromises quality of care																														
2.7.1. Hardships for patients and families, compromised safety and occurrence of serious incidents		2												3	2				**7**										**0**	**7**
2.7.2. Severe emotional and physical harm to patients, families and communities	1																		**1**										**0**	**1**
2.8. Increase in involuntary admissions due to lack of timely voluntary admission at an earlier stage of illness														2					**2**										**0**	**2**
2.9. Increasing suicide rates	1		1												2				**4**										**0**	**4**
2.10. Sub-groups of people with severe mental illnesses are still in need of psychiatric inpatient beds											1			1	3			1	**6**										**0**	**6**
2.10.1. Need for the development of safe, modern and humane asylums that provide long-term residential care for people with severe mental illnesses															2				**2**										**0**	**2**
2.10.2. Lack of available inpatient beds and treatment for schizophrenia patients			1														1		**2**										**0**	**2**
2.11. Insufficient and ineffective community services	1		1					1					1		3				**7**	2									**2**	**9**
2.11.1. Limited post-discharge support in the community														4					**4**										**0**	**4**
2.11.2. Long waiting lists for outpatient services															1	1			**2**										**0**	**2**
2.11.3. Implementation of community care complements, but does not replace inpatient care	1		1										1	3					**6**										**0**	**6**
2.12. Lack of affordable and supported housing services																														
2.12.1. Discharge to homelessness and shelters		1												1	1			1	**4**										**0**	**4**
2.13. Criminalization of mentally ill			1				1						1	2	4				**9**									**2**	**2**	**11**
2.13.1. Increasing detention rates due to lack of adequate and timely mental health treatments of persons with severe mental illnesses (and comorbid substance use disorders)	1													2	2				**5**										**0**	**5**
2.13.2. Delays in transferring individuals with mental disorders in the criminal justice system to hospitals due to inpatient bed shortage														1					**1**										**0**	**1**
Total	10	5	9	1	0	2	2	1	1	0	1	1	3	34	34	1	1	7	**113**	**2**	**1**	**1**	**2**	**1**	**0**	**1**	**2**	**2**	**10**	**123**

*NZ, New Zealand; UK, United Kingdom; US, United States of America; HIC, High- and upper-middle income countries; LMIC, Low- and Middle-Income countries*.

### Extracted Themes

We distinguished two main categories: (a) arguments to reduce psychiatric bed numbers, and (b) arguments to maintain or increase psychiatric bed numbers. In both categories, we identified themes and subthemes supporting each trend (Annex 1). We identified 36 quotes arguing for a decrease and 123 quotes supporting to increase or maintain psychiatric bed numbers.

**Figure 2:**
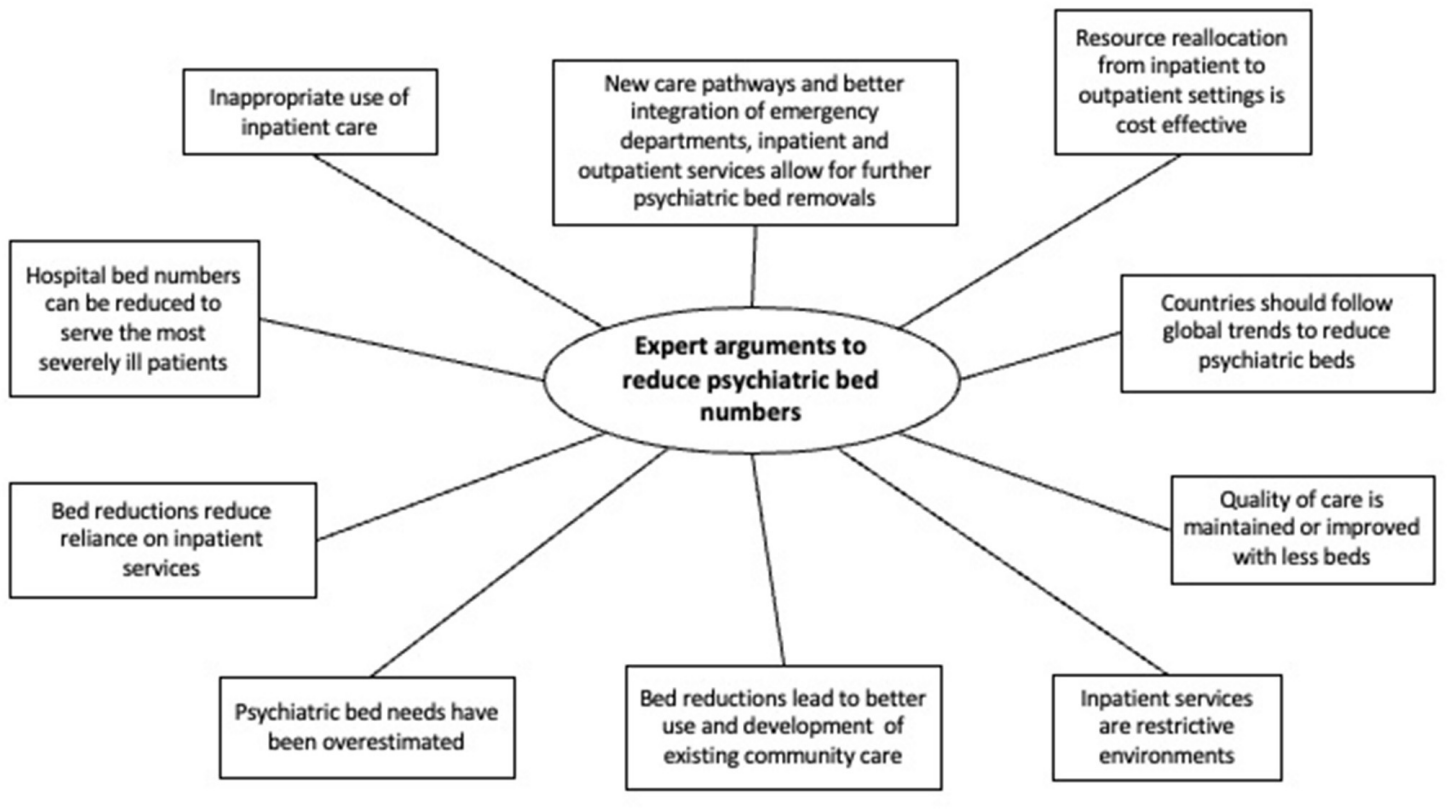
Expert arguments to reduce psychiatric bed numbers, a systematic review of qualitative data.

**Figure 3:**
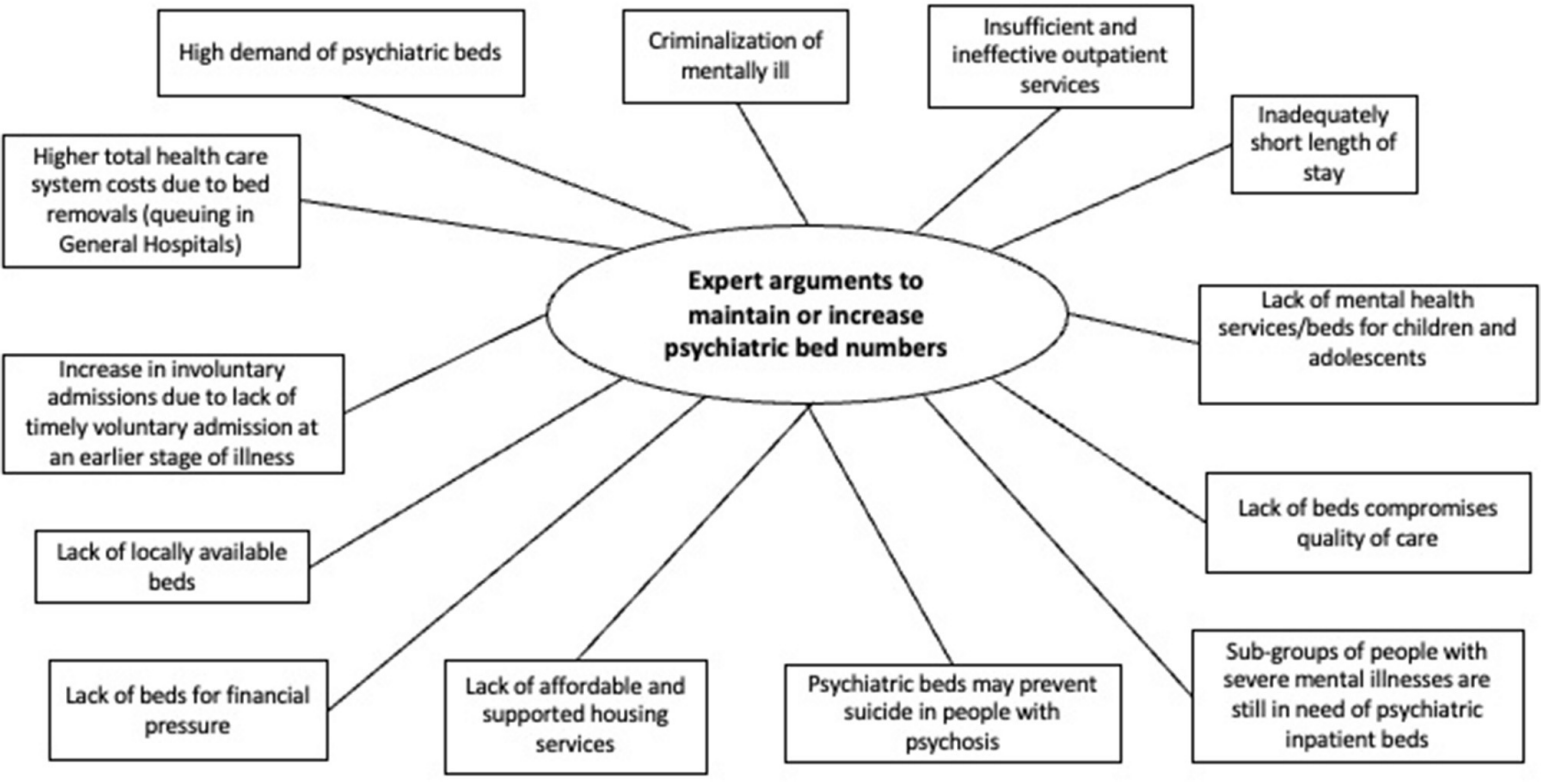
Expert arguments to maintain or increase psychiatric bed numbers, a systematic review of qualitative data.

Illustrative verbatim are given in Table 3. A thematic map was built in order to show the main themes that emerged from the data (Figures 2, 3), which are described in further detail below.

**Table 3:** Expert arguments for trends of psychiatric bed numbers: themes, subthemes and verbatim.

**Category**	**Theme**	**Subtheme**	**Verbatim**
Expert arguments to reduce psychiatric bed numbers	1. Resource reallocation from inpatient to outpatient settings is cost effective	Lower cost of home treatment and outpatient care per individual	“The combination of adding a home treatment team and halving the number of inpatient beds was, when compared to a control area, associated with (a) additional numbers of people receiving acute care (b) a lower cost per individual and (c) no difference in overall service cost” (37)
		Implementation of day hospital services and home treatment teams allow for greater concentration of inpatient resources on most severely ill patients, leading to cost savings	“The major reduction in the number of acute inpatient beds and the opening of an acute day hospital resulted in greater concentration of inpatient resources on the more severely ill patients” (64)
		Reduce resources for inpatient care to develop outpatient care	“The precarious extra-hospital network has been used as a barrier to deactivation of psychiatric beds, although the latter generates the necessary resources for the former” (104)
	2. Inappropriate use of inpatient care	Inappropriately long psychiatric inpatient care	“36% of patients do not need to be in hospital if appropriate after-care could be found” (33)
		Reduced number of long-stay patients allows for further bed removals	“Alternatives to the mental hospital exist and may limit the use of long stay hospital beds through comprehensive community care that also includes proper residential provisions” (81)
		Inpatient psychiatric bed capacity and availability generates utilization and coercive treatments	“Substantially lower rates of bed provision than those currently provided, with the concomitant development of a wide range of community based services could do much to prevent the current excessive tendency to commit patients and to the fostering of disability and dependency which perpetuates the continuing need for such beds” (57)
		Economic incentives for inadequately long inpatient bed use	“It will be necessary in the future to transit from a medical fee system that promotes long-term hospitalization and large-scale expansion to one in which downsizing correlates with better financial results” (116)
	3. Bed reductions lead to better use of existing community care		“Discharge of new long stay patients within a psychiatric service that is community-oriented, support patients in their own homes and make the fullest possible use of non-hospital residential and nursing homes” (79)
	4. Quality of care is maintained or improved with less beds	Bed reductions, while maintaining personnel, improves inpatient care conditions	“The reduction in the number of beds in the public psychiatric sector has led to significant improvement in nursing conditions” (82)
		Bed reductions do not affect the quality of care in the system as a whole and have not shown negative effects	“A 50% reduction in acute beds—and a 23% reduction in total beds—on an inpatient service that had been operating at full capacity was not associated with anticipated negative effects, such as increased demand for psychiatric emergency services, decreased access to emergency or inpatient services, increased recidivism to inpatient care, or increased levels of inadequately treated mental illness in the community” (6)
	5. Psychiatric bed needs have been overestimated	Trend analyses show less psychiatric bed needs of schizophrenia patients	“Present results showing a reduction of the number of schizophrenic inpatients to two-fifths of the present number is significant for hospital planning and healthcare resource allocation” (117)
		Decrease in first-ever admission rates of schizophrenia	“First-ever admission rates of schizophrenia in Denmark have decreased since 1970. The most obvious explanation is the extensive restructuring of the psychiatric service of which a decrease in available beds of more than 50% seems to be most important” (109)
		Low inpatient occupancy rates	“The most apparent consequence of this ineffective planning is that many centers have more beds than they require. A third of the centers in our sample had occupancy rates of 50% or less” (95)
	6. Inpatient services are restrictive environments		“We conclude that greater emphasis and urgency needs to be placed on moving patients on from acute mental health units after 9 weeks of admission. This can lead to more appropriate care for patients in less restrictive environments and reduce demand on acute psychiatric units and reduce the necessity and stress to patients and careers of acute admissions far from home” (48)
	7. New care pathways and better integration of emergency departments, inpatient and outpatient services allow for further psychiatric bed removals		“Our study demonstrated that reducing beds and introducing new care pathway interventions in inpatient and community settings are associated with better ward practices and improvements in patient flow between the emergency department, the inpatient ward and community teams” (5)
	8. Countries should follow global trends to reduce psychiatric beds		“In Korea, however, admission remains the foremost resource in psychiatric treatment. In contrast with the general trend in most developed countries, the number of psychiatric beds in Korea has continuously increased, and the length of stay of psychiatric patients in Korea has remained long for years” (80)
	9. Bed reductions reduce reliance on inpatient services		“Thus increasing psychiatric bed provision would, in the current climate of scarcity, be both profligate and pointless. Let us instead dedicate the limited resources we have to improving the quality of existing inpatient services and increasing their acceptability to patients, and to implementing as fully as we can the knowledge that we already have about how reliance on inpatient services may be reduced” (100)
	10. Hospital bed numbers can be reduced to serve the most severely ill patients		“Our data suggest that an ever broader spectrum of persons with severe mental illness can be managed in the community as more community-based and alternative inpatient settings are created to meet their needs. But the most difficult populations remain, and they appear resistant to permanent exclusion from the state hospital, even in the best-funded community systems” (90)
Expert arguments to maintain or increase psychiatric bed numbers	1. Lack of beds for financial pressure	Financial pressure on the mental health system has resulted in too many bed removals and underfunded inpatient care systems	“There is a risk that the significant financial pressures on mental health trusts can result in too many bed closures” (45)
		Financial disincentives and unfair reimbursement practice have led to lower numbers of psychiatric beds than actually needed	“Specific emphasis should be placed on lobbying for fair reimbursement of services, including psychiatric emergency and inpatient services, as care places a financial strain on hospitals, thus providing a disincentive for hospitals to keep units open or add to existing services” (63)
	2. Higher total health care costs due to bed removals (queuing in General Hospitals)		“Despite the decreased number of extended care days and the increased supply of residential care slots, individuals having acute care episodes that required hospitalization had higher episode and annual costs in the post (state hospital) closure period. The data suggest that the increased costs were due primarily to the increased use of acute care general hospital days that were the consequence of patients queuing up in general hospitals while waiting for a transfer to an intermediate care unit” (115)
	3. High demand of psychiatric beds	High occupancy rates and overcrowding	“In addition, many in-patient wards now regularly have a 100–120% occupancy rate, which is significantly higher than the 85% recommended by the Royal College of Psychiatrists” (87)
		Increasing admission rates and waiting times	“Findings of the study indicate that psychiatric admissions in psychiatric ward are increasing year after year” (68)
		Overcrowding and long waiting times in emergency departments	“(Psychiatric) boarding has occurred for many years in the shadows of mental health care as both inpatient beds and community services have decreased” (27)
	4. Inadequately short length of stay	Short length of stay and premature discharge	“The average length of stay, varying from less than a week in the USA to 15 days in the UK, is inadequate for adequate assessment or treatment. …hospital managers spend a large proportion of their time juggling the relative risks of discharging patients prematurely or delaying admission” (73)
		Revolving door effect: Early readmission rates	“Length of stay and the crisis discharge policy seem to exacerbate the revolving door effect in this psychiatric hospital. Readmission is often used as quality indicator for inpatient psychiatric services, and could be seen as a failure of the earlier hospital admission” (62)
	5. Lack of specialized psychiatric beds for children and adolescents		“In Wales no psychiatrist has access to an adolescent psychiatric in-patient bed for emergency admissions” (31)
	6. Lack of locally available beds	Need for the development of integrated health care systems with decentralized inpatient care capacities	“In broad terms, the study recommends an increase in the number of acute psychiatric beds in general hospitals; development of community-based residential care; redistribution of staff from hospital to community services, particularly in rural areas; and the development of information systems to monitor the transitions to community-based care” (55)
		Risk of transfer outside patients' local community for care	“Inadequate local and regional psychiatric hospital (bed) capacity results in significantly prolonged emergency department length of stay and puts many patients at risk for transfer outside their local community for care” (66)
	7. Lack of beds compromises quality of care	Hardships for patients and families, compromised safety and occurrence of serious incidents	“Demoralization of patients and staff, with premature discharges and patients being placed inappropriately in isolating bed and breakfast or hostel accommodation with untrained or ill prepared staff. Under such circumstances, conditions are ripe for the occurrence of serious incidents” (97)
		Severe emotional and physical harm to patients, families and communities	“If a person in need is unable to access an acute bed, severe emotional or at times physical harm to them and their career or family is a potential or high risk and can affect the wider community” (125)
	8. Increase in involuntary admissions due to lack of timely voluntary admission at an earlier stage of illness		“The reduction in beds has been matched by a parallel, >60% increase in involuntary admissions during the same period, which does not seem to be matched by an increase in national mental health disorders and is possibly related to increase symptom severity at the time of presentation” (87)
	9. Psychiatric beds may prevent suicide in people with psychosis		“There has been a 20% increase in Australian suicide rates over the decade 2006–2016” (29)
	10. Sub-groups of people with severe mental illnesses are still in need of psychiatric inpatient beds	“The lack of change in bed use supports the view that there is a 'bed-rock' of serious illness which will always need in-patient care” (106)	
		Need for the development of safe, modern and humane asylums that provide long-term residential care for people with severe mental illnesses	“This was the original meaning of psychiatric “asylum” —a protected place where safety, sanctuary, and long-term care for the mentally ill would be provided. It is time to build them—again” (84)
		Lack of available inpatient beds and treatment for schizophrenia patients	“This population-based investigation showed an increase over time in the number and proportion of patients with schizophrenia who were not discovered until many days after death, which was correlated with the decrease in the number of available hospital beds for this group of patients” (110)
	11. Insufficient and ineffective community services	“The monies saved in closing psychiatric institutions and moving (too few) beds into the general hospitals were to be redirected to effective community programmes, but this has largely not occurred” (29)	
		Limited post-discharge support in the community	“This could indicate that hospitals are allocating scarce beds to the most vulnerable patients, or that it is more challenging to accelerate the discharges of older patients, for example due to limitations in the availability of post-discharge support in the community” (38)
		Long waiting lists for outpatient services	“The waiting time for outpatient psychiatric treatment in the central Slovenian region has been increasing, presently being 4 months on average. The access to psychiatric outpatient facilities, which used to be easy in the past even without referral forms, is becoming now increasingly difficult” (118)
		Implementation of community care complements, but does not replace inpatient care	“Our brief review of the literature on community based residential alternatives to acute psychiatric care suggests that these services are not alternatives for all patients, and as such are not completely substitutable for acute care” (67)
	12. Lack of affordable and supported housing services	Discharge to homelessness and shelters	“Discharge from psychiatric wards to shelters or the streets is a real problem “/” Practitioners need to recognize that a shelter is not an appropriate ‘address’ for discharging individuals recovering from mental illness.” (91)
	13. Criminalization of mentally ill	“(…) the shortage of public psychiatric beds contributes to a number of costly and sometimes dangerous social problems, including jails and prisons overcrowded with inmates who are acutely ill and untreated” (119)	
		Increasing detention rates due to lack of adequate and timely mental health treatments of persons with severe mental illnesses (and comorbid substance use disorders)	“Funding more psychiatric beds would reduce the detention rates by allowing timely voluntary admission to a local acute psychiatric bed at an earlier stage of illness” (85)
		Delays in transferring individuals with mental disorders in the criminal justice system to hospitals due to inpatient bed shortage	“More secure psychiatric beds may be required, at least in the short term, to support diversion policies and enable compliance with national policy directive, and to establish whether redesigned pathways can enhance treatment and behavioral outcomes for acutely mentally ill prisoners on a larger scale” (92)

#### Expert Arguments to Reduce Psychiatric Bed Numbers

Ten themes emerged as arguments supporting a trend to reduce psychiatric bed numbers.

##### Inpatient Care Has Inappropriately Long Duration

“36% of patients do not need to be in hospital if appropriate after-care could be found” (33), which often leads to an inappropriately long use of inpatient care. Reducing long-stay patients allows for further bed removals, especially considering that inpatient bed capacity and availability generates utilization and coercive treatments. However, there are economic incentives for inadequately long inpatient bed use (39, 40, 48, 57, 64, 71, 78, 79, 81, 83, 116).

##### New Care Pathways With Better Integration of Emergency Departments, Inpatient and Outpatient Services Allow for Further Psychiatric Bed Removals

Developing an integrated and well-balanced health care system combining acute and community-based care has shown improved outcomes, such as better ward practices and improvements in patient flow between the different mental health services (5, 39, 88).

##### Resource Reallocation From Inpatient to Outpatient Settings Is Cost Effective

Better financial and treatment outcomes can be achieved by reallocating resources within the mental health system, as the cost of home-based treatments is lower. Resources reallocated from inpatient services can be used to develop outpatient care. The implementation of day hospital services and home treatment teams allows for a greater concentration of inpatient resources on the most severely ill patients, leading to cost savings (40, 57, 64, 104). “The combination of adding a home treatment team and halving the number of inpatient beds was, when compared to a control area, associated with (a) additional numbers of people receiving acute care (b) a lower cost per individual and (c) no difference in overall service cost” (37).

##### Countries Should Follow Global Trends to Reduce Psychiatric Beds

In several countries with comparatively high numbers of psychiatric beds or high reliance on psychiatric beds within the service system as in Korea, Japan and Moldova, the call to follow global trends of psychiatric bed reductions has been made (80, 83, 88).

##### Quality of Care Is Maintained or Improved With Less Beds

No anticipated negative effects have been associated with bed reductions. On the contrary, bed reductions, while maintaining personnel, improves inpatient care conditions. Statements included decongestion of wards, appropriate staffing and training and development of additional programs contributing to better treatment (6, 28, 82).

##### Inpatient Services Are Restrictive Environments

The transfer of patients from acute to community care allows for treatment in a more adequate setting, which could improve aggressive behavior and reduces stress of patients and careers generated by acute admissions far from home (48, 120).

##### Bed Reductions Lead to Better Use of Existing Community Care

Reducing long-term hospitalization foments treatments outside of hospital facilities, possibly in their own homes (79).

##### Psychiatric Bed Needs Have Been Overestimated

Ineffective planning of beds and lack of understanding of acute care has led to an overestimation for the number of beds needed. Statements included low occupancy rates, and trend analyses showing less psychiatric bed need and a decrease in first-ever admission rates of schizophrenia patients (95, 109, 117).

##### Bed Reductions Reduce Reliance on Inpatient Services

Increasing psychiatric bed numbers would be pointless as bed reductions reduce dependence on inpatient care. There is a need to focus scarce resources on improving the quality of care of existing inpatient services (100).

##### Hospital Bed Numbers Should Be Reduced to Serve the Most Severely Ill Patients

Inpatient care should focus on acute care for the most severely ill patients. However, inpatient psychiatric beds should not be eliminated as this subgroup cannot be successfully treated in the community (90).

#### Expert Arguments to Maintain or Increase Psychiatric Bed Numbers

Regarding the second category of expert arguments to maintain or increase psychiatric bed numbers, thirteen themes emerged.

##### High Demand of Psychiatric Beds

Deinstitutionalization has resulted in increased occupancy rates and overcrowding, frequently over 100%, along with increasing admission rates and waiting times in both HICs and LMICs (10, 25, 27, 30, 32, 36, 41, 43, 44, 46, 49, 50, 52, 54, 56, 59, 60, 65, 66, 68, 69, 74, 87, 99, 103, 114, 119, 122, 126). “Mental health services in Australia, Canada, the UK, and the USA are all struggling to meet the demand for bedded care and are experiencing negative outcomes such as out of catchment admissions, access blocks in emergency departments, excessively high ward occupancy, discharge to homelessness, criminalization of the mentally ill, and early readmission” (10).

##### Criminalization of Mentally Ill

Increasing detention rates result from a lack of adequate and timely mental health treatment of persons with severe mental illnesses (and comorbid substance use disorders), as well as delays in transferring individuals with mental disorders in the criminal justice system to hospitals due to bed shortage (8, 10, 47, 59, 70, 75, 85, 92, 93, 96, 102, 105, 107, 119, 124). One publication reports: “Because of the lack of accessible and effective services for those with serious mental illnesses, patients are falling through the net of psychiatric services and are imprisoned for minor public order offenses” (113).

##### Insufficient and Ineffective Community Services

There has been a wide gap between the closure of inpatient beds and the development of alternative care, which has resulted in limited post-discharge support in the community and long waiting lists for outpatient services. One study mentions: “The monies saved in closing psychiatric institutions and moving (too few) beds into the general hospitals were to be redirected to effective community programmes, but this has largely not occurred” (29). In addition, the implementation of community care complements, but does not replace inpatient care. Even where decentralized services have been developed, it still appears that there are too few inpatient beds to adequately treat acutely-ill patients (9, 14, 34, 35, 38, 42, 50, 52, 53, 59, 67, 72, 73, 87, 106, 108, 111, 118, 123).

##### Inadequately Short Length of Stay

Premature discharges have led to early readmission rates, frequently referred to as the “revolving door effect”, which often results in patients being inappropriately placed in extra-hospital accommodation with untrained staff (10, 25, 38, 62, 72, 73, 86, 91, 97, 99, 101, 107).

##### Lack of Specialized Psychiatric Beds for Children and Adolescents

Mental health professionals have to “borrow” beds from adult services or considerably delay treatment, which in turn leads to increased emotional disorders, suicide rates, drug overdose and criminalization of young people with mental illness (31, 51, 72, 93, 94, 98, 122).

##### Lack of Beds Compromises Quality of Care

Significant hardships for patients and families compromises their safety and increases the probability of the occurrence of serious incidents, along with severe emotional and physical harm to them and their families (26, 27, 51, 65, 66, 73, 125). One publication reports: “Demoralization of patients and staff, with premature discharges and patients being placed inappropriately in isolating bed and breakfast or hostel accommodation with untrained or ill prepared staff. Under such circumstances, conditions are ripe for the occurrence of serious incidents” (97).

##### Sub-Groups of People With Severe Mental Illnesses Are Still in Need of Psychiatric Inpatient Beds

This small group of patients have shown an inability to be treated in non-acute settings. In addition, there is a need to develop safe, modern and humane asylums that provide long-term residential care for the severely mentally ill (47, 70, 77, 84, 86, 99, 110, 119). One study reports: “The lack of change in bed use supports the view that there is a 'bed-rock' of serious illness which will always need inpatient care” (106).

##### Psychiatric Beds May Prevent Suicide in People With Psychosis

There has been an increase of suicide rates between 20 and 100% in people with non-organic psychosis. Providing psychiatric beds during crisis may prevent suicide in this population (29, 59, 93, 123).

##### Lack of Affordable and Supported Housing Services

Delayed admissions and early discharges lead to patients being discharged to homelessness, as they cycle through emergency departments, shelters and criminal settings without receiving adequate treatment (10, 36, 91, 119).

##### Lack of Beds for Financial Pressure

Financial disincentives and unfair reimbursement practices have led to lower numbers of psychiatric beds than actually needed (49, 63). “There is a risk that the significant financial pressures on mental health trusts can result in too many bed closures” (45).

##### Lack of Locally Available Beds

There is a need to decentralize inpatient services from urban to rural areas, as this uneven distribution of beds has resulted in prolonged lengths of stay in emergency departments, along with a higher risk of transfer outside of patients' community for care leading to significant hardships as they have to travel long distances to access treatment (55, 66, 73, 76).

##### Increase in Involuntary Admissions Due to Lack of Timely Voluntary Admission at an Earlier Stage of Illness

Delayed admissions and premature discharges end up in involuntary longer lengths of stay possibly due to increased symptom severity at intake (73, 87).

##### Higher Total Health Care System Costs Due to Bed Removals (Queuing in General Hospitals)

Patients in acute care who are waiting for transfers to an intermediate care unit are generating bed blocks in general hospitals, thus increasing total costs (115).

## Discussion

### Main Findings

This systematic review on expert arguments regarding trends of psychiatric bed numbers was based on 106 publications from 25 countries, including 14 studies from LMICs. First, there was not any general agreement on the direction of trends that should be pursued in policies, as there were arguments for reductions as well as for increases of psychiatric bed rates. Secondly, a complex matrix of partially contradicting arguments emerged, i.e., with respect to costs and quality of care. In the absence of hard evidence, those arguments remain relevant and have to be carefully considered in specific contexts for local policies.

### Comparison With the Literature

Mental health service planners need orientation on how to further develop the psychiatric inpatient sector (7–10). Despite the diversity of services and nomenclatures used across regions (10, 16–18), there was agreement on several themes, such as the need to develop integrated mental health systems that assure coherence and continuity of care, along with the need to aim for specific populations and contexts (127). In all, arguments expressing concern about further bed reductions prevailed as there is a high demand on inpatient services, especially on short-stay and acute beds, evidenced in high occupancy and increasing admission rates (10, 99). In the US and Australia, overcrowding and long waiting times in emergency departments showed to be particularly relevant (25, 27, 30, 43, 52, 60, 66, 119). The relevance of psychiatric beds for problems that arise outside of the health system, such as violent crime of people with mental illnesses, increased detention rates and discharge to homelessness due to a lack of timely treatment and admission, was considered in several HICs (36, 61, 85, 128). Criminalization of mentally ill consistently appeared as a relevant theme in the US (10, 70, 93, 119) and was also mentioned for Latin America (8, 105). Inadequately short lengths of stay emerged as an issue in HICs (73), and in South Africa among LMICs (62). Mental health professionals are forced to prematurely discharge patients in order to free already scarce beds risking short readmission intervals, referred to as the revolving door effect (99). Several authors have expressed their concern that there are gaps in the mental health system due to major reductions of inpatient bed capacities that have been implemented without the appropriate development of community care (52, 129). There was agreement for HICs and LMICs that insufficient provision of outpatient services may not compensate for more bed reductions, resulting in overcrowding of inpatient facilities, which subsequently compromises the quality care (99). Several authors therefore call to halt current trends to further remove beds. Within this context, limited post discharge support in the community showed to be an important point in the UK (14, 38, 50, 87), and the lack of specialized psychiatric beds for children and adolescents emerged as particularly relevant in the UK and the US. Other subspecialty beds, such as mother baby units, may also be lacking, especially in LMICs (130). This overall lack of beds compromises patient safety and quality of care, carrying hardships for patients and families, and may provoke serious incidents (97). Some arguments emerged for specific contexts, such as increasing suicide rates related to major bed reductions, especially in the US (93, 123), and the argument that financial pressures on the mental health system have resulted in too many bed removals in Greece and the UK (45, 49).

In regard to arguments supporting the further reduction of psychiatric beds, most authors referred to long-stay beds and to inappropriately long lengths of stay (71, 78, 79, 81, 83), emphasizing that patients could be transferred to the community if more timely and adequate after-care could be found (33, 39, 40, 48, 64). The paradigm emerged that successful development of effective community services, including housing, and a better integrated mental health system would allow to continue psychiatric bed removals without negative outcomes (4–6), especially considering that inpatient services can be restrictive environments (48). We observed that a further reduction of psychiatric beds was usually not recommended for LMICs, except for Moldova (88). Several authors identified a need to follow trends of psychiatric bed reductions, which were enforced in most developed countries. This was mentioned for Japan (83) and Korea (80). For New Zealand authors argued that inpatient psychiatric bed capacity and availability generates utilization and coercive treatments (57). Economic incentives for inadequately long inpatient bed use have been observed in Japan (116). In the UK, it was suggested that bed reductions lead to better use and development of existing community care, along with reduced reliance on such beds (100). Lastly, for the US it is suggested that hospital bed numbers should be reduced to serve the most severely ill patients (90).

Opinions were divided on themes, such as the cost-effectiveness of psychiatric bed reductions. On the one hand, the lower cost of community services compared to inpatient care allows for greater concentration of inpatient resources on the most severely ill. However, in 1998 an US-American study showed increased costs in the entire health system after bed reductions due to a higher use of acute care in general hospitals as a result of severely mentally ill patients queuing up in these facilities while waiting for transfers to intermediate care in the community (115). In regard to quality of care opinions were also divided. Lack of beds can lead to reduced quality of inpatient treatments and overcrowding. However, hospitals in the US have shown to maintain or increase their quality of care by removing beds while maintaining the staff (82).

Our study shows that an empirical approach to argue for trends of psychiatric bed numbers was most frequent, while the normative approach was least frequently used with no differences between HICs and LMICs. It has been argued that need of psychiatric beds may vary between regions (131), and even changes between seasons (132). The provision of psychiatric beds has to respond to local requirements and conditions (127). The arguments presented here may help to tailor policies after evaluation of fit and context-dependent applicability. One-dimensional models that simply focus on the numbers to address possible bed shortages may need to consider incorporating more complex aspects of the system, including costs, quality and pathways of care (133). At the level of catchment areas, the need of beds has to be evaluated assuring a continuum of care with quality treatment before, during and after an acute episode of mental illness (134).

### Strengths and Limitations

To our knowledge this is the first systematic review of expert arguments on psychiatric bed numbers. We provide evidence and examined arguments from 25 countries worldwide, including 14 LMICs. This research has several limitations. The arguments in the present review were not necessarily identical to the main objective of the respective studies but were extracted from the discussions during our screening process. Secondly, the usage of variable nomenclatures referring to psychiatric beds is a further limitation. Thirdly, study quality was not assessed. Another limitation was that this review did not assess the opinions of patients, caregivers, families and other members of the community.

### Conclusion

Several implications arise from our findings. First, there are relatively few arguments that are repeated in the literature and should be considered by discussants on the required number of psychiatric beds. This synthesis of arguments can help to focus future debate and to guide policymakers who need to define targets for the number of psychiatric beds in specific countries and catchment areas. Secondly, further research is needed to guide which arguments are best suited for specific contexts. Thirdly, there is need to establish targets for more specific populations (e.g., juvenile, older adult, forensic, acute and long-stay populations) and for specific types of facilities (public, private, mental or general hospital and residential). The low number of publications from LMICs highlights the need for further evidence from these countries, especially from regions underrepresented in research, such as Central and East Asia, Africa and Central America.

## Data Availability Statement

The original contributions presented in the study are included in the article/supplementary material, further inquiries can be directed to the corresponding author/s.

## Author Contributions

Literature screening was conducted by SD, ER, and MS. Data were extracted independently by SD, ER, and MS. SD and AM performed the data analysis. ER and MS reviewed it. SD wrote the manuscript. AM and SP revised and corrected it along the process. All authors contributed to the article and approved the submitted version.

## Funding

This systematic review was funded by the Agencia Nacional de Investigación y Desarrollo in Chile, grant scheme FONDECYT Regular, Grant No. 1190613.

## Conflict of Interest

The authors declare that the research was conducted in the absence of any commercial or financial relationships that could be construed as a potential conflict of interest.

## Publisher's Note

All claims expressed in this article are solely those of the authors and do not necessarily represent those of their affiliated organizations, or those of the publisher, the editors and the reviewers. Any product that may be evaluated in this article, or claim that may be made by its manufacturer, is not guaranteed or endorsed by the publisher.
