# Corrigendum: Expert arguments for trends of psychiatric bed numbers: A systematic review of qualitative data

**DOI:** 10.3389/fpsyt.2022.957272

**Published:** 2022-08-02

**Authors:** Adrian P. Mundt, Sabine Delhey Langerfeldt, Enzo Rozas Serri, Mathias Siebenförcher, Stefan Priebe

**Affiliations:** ^1^Medical Faculty, Universidad Diego Portales, Santiago, Chile; ^2^Departamento de Neurología y Psiquiatría, Clínica Alemana de Santiago, Facultad de Medicina Clínica Alemana, Universidad del Desarrollo, Santiago, Chile; ^3^Department of Psychiatry and Mental Health, Hospital Clínico Universidad de Chile, Santiago, Chile; ^4^Department of Psychiatry and Psychotherapy Campus Mitte, Charité Universitätsmedizin Berlin, Berlin, Germany; ^5^Unit for Social and Community Psychiatry (WHO Collaborating Centre for Mental Health Service Development), Queen Mary University of London, London, United Kingdom

**Keywords:** psychiatric hospital beds, general hospital psychiatry, institutionalization, expert recommendation, consensus, inpatient, length of stay

In the original article, there was a mistake in Table 2 as published.

**Table 2:** Number of expert arguments per theme and country.

**Themes**	**World Bank Income classification**																													
	**High-Income Countries (HIC)**																			**Low- and Middle-Income Countries (LMIC)**										
	**Australia**	**Canada**	**Denmark**	**Finland**	**Greece**	**Hong Kong**	**Ireland**	**Italy**	**Japan**	**Korea**	**Netherlands**	**NZ**	**Norway**	**UK**	**US**	**Slovenia**	**Sweden**	**Global**	**Total HIC**	**Brazil**	**Buthan**	**China**	**Ghana**	**Malawi**	**Moldova**	**Uganda**	**South Africa**	**South America**	**Total LMIC**	**Total HIC and LMIC**
**Expert arguments to reduce psychiatric bed numbers**																														
1.1. Cost effectiveness																														
1.1.1. Lower overall cost of home-based treatment compared with inpatient services												1		1	1				**3**										**0**	**3**
1.1.2. Implementation of a day hospital service and home treatment teams allows for greater concentration of inpatient resources on most severely ill patients, leading to cost savings							1												**1**										**0**	**1**
1.1.3. Reduce resources for inpatient care to develop outpatient care																			**0**	1									**1**	**1**
1.2. Inappropriate use of inpatient care																														
1.2.1. Inappropriately long psychiatric inpatient care							1		1					3	1				**6**										**0**	**6**
1.2.2. Reduced number of long-stay patients allows for further psychiatric bed removals		1							1					2				1	**5**										**0**	**5**
1.2.3. Inpatient psychiatric bed capacity and availability generates utilization and coercive treatments												1							**1**										**0**	**1**
1.2.4. Economic incentives for inadequately long inpatient bed use									1										**1**										**0**	**1**
1.3. Bed reductions lead to better use and development of existing community care														1					**1**										**0**	**1**
1.4. Quality of care is maintained or improved with less beds																														
1.4.1. Bed reductions, while maintaining personnel, improves inpatient care conditions					1														**1**										**0**	**1**
1.4.2. Bed reductions do not affect the quality of care in the system as a whole and has not shown negative effects															2				**2**										**0**	**2**
1.5. Less psychiatric bed needs																														
1.5.1. Trend analyses show less psychiatric bed needs of schizophrenia patients									1										**1**										**0**	**1**
1.5.2. Decrease in first-ever admission rates of schizophrenia			1																**1**										**0**	**1**
1.5.3. Low inpatient occupancy rates															1				**1**										**0**	**1**
1.6. Inpatient services are restrictive environments														2					**2**										**0**	**2**
1.7. New care pathways and better integration of emergency departments, inpatient and outpatient services allow for further psychiatric bed removals	1							1						1					**3**						1				**1**	**4**
1.8. Follow global trends of psychiatric bed reductions in most of the developed countries									1	1									**2**						1				**1**	**3**
1.9. Bed reductions reduce reliance on inpatient services														1					**1**										**0**	**1**
1.10. Hospital bed numbers should be reduced to serve the most severely ill patients															1				**1**										**0**	**1**
Total	**1**	**1**	**1**	**0**	**1**	**0**	**2**	**1**	**5**	**1**	**0**	**2**	**0**	**11**	**6**	**0**	**0**	**1**	**33**	**1**	**0**	**0**	**0**	**0**	**2**	**0**	**0**	0	**3**	**36**
**Expert arguments to increase or maintain psychiatric bed numbers**																														
2.1. Lack of beds for financial pressure																														
2.1.1. Financial pressure on the mental health system has resulted in too many bed removals and underfunded inpatient care systems						1								1					**2**										**0**	**2**
2.1.2. Financial disincentives and unfair reimbursement practice have led to lower numbers of psychiatric beds than actually needed															1				**1**										**0**	**1**
2.2. Higher total health care system costs due to bed closures (queuing in General Hospitals)															1				**1**										**0**	**1**
2.3. High demand of psychiatric beds																														
2.3.1. High occupancy rates and overcrowding			1			1	1		1			1		2				1	**8**				2			1			**3**	**11**
2.3.2. Increasing admission rates and waiting times	1	1	2											3	1				**8**		1								**1**	**9**
2.3.3. Overcrowding and long waiting times in emergency departments	3													1	5			1	**10**										**0**	**10**
2.4. Inadequately short length of stay																														
2.4.1. Short length of stay and premature discharge		1		1										3	2				**7**										**0**	**7**
2.4.2. Revolving door effect: Early readmission rates	1		1											1				1	**4**								1		**1**	**5**
2.5. Lack of specialized psychiatric beds for children and adolescents														3	3				**6**			1							**1**	**7**
2.6. Lack of locally available beds																														
2.6.1. Need for the development of integrated health care systems with decentralized inpatient care capacities																			**0**					1			1		**2**	**2**
2.6.2. Risk of transfer outside patients' local community for care														1	1				**2**										**0**	**2**
2.7. Lack of beds compromises quality of care																														
2.7.1. Hardships for patients and families, compromised safety and occurrence of serious incidents		2												3	2				**7**										**0**	**7**
2.7.2. Severe emotional and physical harm to patients, families and communities	1																		**1**										**0**	**1**
2.8. Increase in involuntary admissions due to lack of timely voluntary admission at an earlier stage of illness														2					**2**										**0**	**2**
2.9. Increasing suicide rates	1		1												2				**4**										**0**	**4**
2.10. Sub-groups of people with severe mental illnesses are still in need of psychiatric inpatient beds											1			1	3			1	**6**										**0**	**6**
2.10.1. Need for the development of safe, modern and humane asylums that provide long-term residential care for people with severe mental illnesses															2				**2**										**0**	**2**
2.10.2. Lack of available inpatient beds and treatment for schizophrenia patients			1														1		**2**										**0**	**2**
2.11. Insufficient and ineffective community services	1		1					1					1		3				**7**	2									**2**	**9**
2.11.1. Limited post-discharge support in the community														4					**4**										**0**	**4**
2.11.2. Long waiting lists for outpatient services															1	1			**2**										**0**	**2**
2.11.3. Implementation of community care complements, but does not replace inpatient care	1		1										1	3					**6**										**0**	**6**
2.12. Lack of affordable and supported housing services																														
2.12.1. Discharge to homelessness and shelters		1												1	1			1	**4**										**0**	**4**
2.13. Criminalization of mentally ill			1				1						1	2	4				**9**									**2**	**2**	**11**
2.13.1. Increasing detention rates due to lack of adequate and timely mental health treatments of persons with severe mental illnesses (and comorbid substance use disorders)	1													2	2				**5**										**0**	**5**
2.13.2. Delays in transferring individuals with mental disorders in the criminal justice system to hospitals due to inpatient bed shortage														1					**1**										**0**	**1**
Total	10	5	9	1	0	2	2	1	1	0	1	1	3	34	34	1	1	7	**113**	**2**	**1**	**1**	**2**	**1**	**0**	**1**	**2**	**2**	**10**	**123**

NZ, New Zealand; UK, United Kingdom; US, United States of America; HIC, High- and upper-middle income countries; LMIC, Low- and Middle-Income countries.

Column headings are labeled as “High- and upper-middle income countries (HIC)“ and ”Lower-Middle and Low-Income countries (LMIC)“. The heading should read ”High-Income Countries (HIC)“ and ”Low- and Middle-Income Countries (LMIC),“ respectively.

Zeros were erroneously inserted in several lines that should have been blank,

On page 12

1.1. Cost effectiveness

1.2 Inappropriate use of inpatient care

1.4. Quality of care is maintained or improved with less beds

1.5. Less psychiatric bed needs

On page 13

Expert arguments to increase or maintain psychiatric bed numbers

2.1 Lack of beds for financial pressure

2.3 High demand of psychiatric beds

2.4 Inadequately short length of stay

2.6. Lack of locally available beds

2.7 Lack of beds compromises quality of care

2.12 Lack of affordable and supported housing services

The numbers for those headings are reported in the subordinate points.

Finally, only in the PDF version of the published article, an error occurs in the heading of the first column in Table 2. The statement “Expert arguments to reduce psychiatric bed numbers,” which is correctly inserted on page 12, is erroneously repeated on pages 13 and 14. Instead, the correct column heading on pages 13 and 14 is “Expert arguments to increase or maintain psychiatric bed numbers.”

The corrected Table 2 is shown below.

The authors apologize for this error and state that this does not change the scientific conclusions of the article in any way. The original article has been updated.

## Publisher's note

All claims expressed in this article are solely those of the authors and do not necessarily represent those of their affiliated organizations, or those of the publisher, the editors and the reviewers. Any product that may be evaluated in this article, or claim that may be made by its manufacturer, is not guaranteed or endorsed by the publisher.

